# Synthesis of 2,3-Dioxo-5-(substituted)arylpyrroles and Their 2-Oxo-5-aryl-3-hydrazone Pyrrolidine Derivatives

**DOI:** 10.3390/molecules14010250

**Published:** 2009-01-07

**Authors:** M.F. Mohammat, Z. Shaameri, A.S. Hamzah

**Affiliations:** Organic Synthesis Laboratory, Institute of Science, Universiti Teknologi MARA (UiTM), 40450 Shah Alam, Selangor, Malaysia

**Keywords:** Pyrrolidine, Hydrazine, Hydrazone, 2,3-Dioxo-5-(substituted)arylpyrroles.

## Abstract

Some novel 2,3-dioxo-5-(substituted)arylpyrroles have been synthesized. Among these, pyrrolidine compound **1b** was converted to 2,3-dioxo-5-aryl pyrrolidine **2b**. Finally a set of hydrazone derivatives was obtained from the reaction of **2b** with various hydrazine salts. The structures of all the new synthesized compounds were confirmed by elemental analyses, IR and ^1^H-NMR spectra.

## Introduction

In connection with our ongoing studies towards the total synthesis of codonopsinine, we became interested in the three-component condensation reaction reported by Dehaen *et al.* [1]. This elegant one pot reaction furnished the important intermediate 2,3-dioxo-5-arypyrroles which are required in our work. Depending on the substitution groups on the aromatic aldehyde used, this reaction will provide different 2,3-dioxo-5-arylpyrroles in reasonable to moderate yields. Particular attention has been made to these classes of compounds since some of the 2,3-dioxo-5-arylpyrroles were successfully converted to their respective 2,3-dioxo-5-arylpyrrolidinones [2]. These pyrrolidinones could be promising intermediates for preparing various synthetically challenging and medicinally important alkaloids such as codonopsinine, anisomycin and preussin [3,4,5]. 

Hydrazine functionalities are also important intermediates for the synthesis of some bioactive compounds such as β-lactams [6]. Furthermore, they have been reported to show a variety of interesting biological activities [7,8,9]. The synthetic versatility of hydrazine has led to the extensive use of this compound in organic synthesis. Although some chemistry of hydrazine and pyrrolidone reactions have been studied [10], to our knowledge reactions of 2,3-dioxo-5-arylpyrrolidinone with different hydrazine salts have not been reported before in open literature. Consequently, new 2,3-dioxo-5-arylpyrrolidinones have now been synthesized and their reactions with various hydrazines are currently being investigated. 

## Results and Discussion

**Scheme 1:**
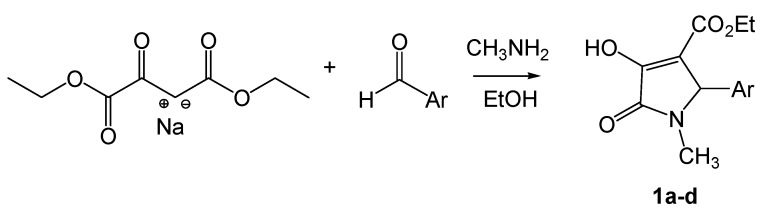
Synthesis of 2,3-dioxo-5-(substituted)arylpyrroles **1a-d**.

Condensation of sodium diethyl oxalacetate with equimolar aldehyde and methylamine in refluxing ethanol gave compounds **1a-d** in a one-pot reaction manner (Scheme 1). Although the yields were moderate, the products could be easily filtered out of the reaction mixture and could be prepared in multigram scale. The structures of the aldehyde components are summarized in Table 1. 

**Table 1:** 2,3-dioxo-5-(substituted)arylpyrroles **1a-1d**.

Compound	Ar
**1a**	Phenyl
**1b**	*p*-OCH_3_ phenyl
**1c**	*p*-CH_3_ phenyl
**1d**	*p*-OH phenyl

**Scheme 2:**
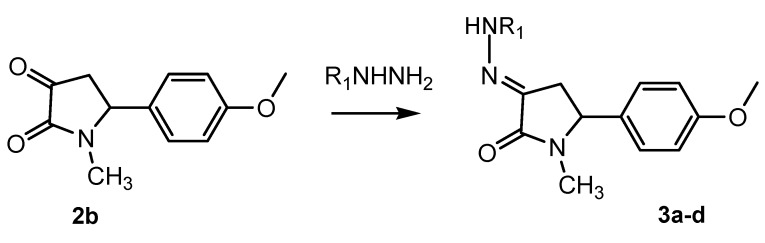
Synthesis of 2-oxo-5-aryl-3-hydrazone pyrrolidine.

2,3-dioxo-5-aryl pyrrolidinone **2b** was successfully prepared by refluxing **1b** in acidic solution for 7 hours [2], after an attempt using a reported method of decarboxylation in refluxing MeCN failed to give the desired product [11,12]. Consequently, the reaction of **2b** with equivalent amounts of various hydrazine salts afforded the corresponding 2-oxo-5-aryl-3-hydrazone pyrrolidine derivatives (Scheme 2). The structures of the hydrazones are summarized in Table 2.

**Table 2:** Hydrazones of **3a-3d**.

Compound	R_1_
**3a**	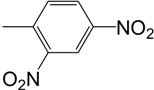
**3b**	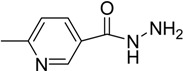
**3c**	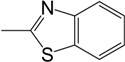
**3d**	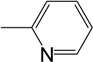

The structures of all new synthesized compounds were confirmed by FTIR, NMR and elemental analysis results. The prominent characteristic of C=O bands of the pyrrolidinone **2b** disappeared and new absorption bands corresponding to the NH group were observed at 3320-3375 cm^-1^ in the IR spectra of all the hydrazone derivatives. New N*H*N=C broad proton singlets at 9.66-11.80 ppm were also observed in all the ^1^H-NMR spectra of all hydrazone derivatives.

## Conclusions

New compounds, 2-oxo-5-aryl-3-hydrazone pyrrolidines, which offer a large number of potential derivatizations have been prepared. The work on the biological activities on these compounds is currently investigated in our laboratory. 

## Experimental

### General

Melting points were determined on a Barnstead Electrothermal melting point apparatus and are uncorrected. ^1^H-NMR and ^13^C-NMR spectra (δ, ppm) were recorded in DMSO-d_6_ solutions on a Varian Unity Inova 300 MHz spectrometer operating in the Fourier transform mode with TMS as internal standard. The IR spectra (υ, cm^-1^) were obtained with a Varian Excalibur 3100. Elemental analyses were performed on a Flash Elemental Analyzer 110 series. All reagents and chemicals were obtained from Aldrich Chemical Company (USA) and Merck and were used as received.

### General procedure for the syntheses of ethyl 4-(hetero) phenyl-2,5-dihydro-1H-pyrrole-3-carboxylates

A suspension of sodium diethyl oxalacetate (1 equiv), 30% methylamine solution in absolute ethanol (1 equiv) and aldehyde (1 equiv) in ethanol was heated at reflux towards complete solution (30 min). After cooling, the mixture was added on ice-water and then acidified with HCl. The precipitate was filtered, washed with water and ether in order to remove traces of aldehyde. After drying under reduced pressure the 2,3-dioxopyrrolidines **1** were obtained with sufficient purity.

*Ethyl 4-hydroxy-1-methyl-5-oxo-2-phenyl-2,5-dihydro-1H-pyrrole-3-carboxylate* (**1a**): Yield 40%, m.p 163-164 °C; ^1^H-NMR: d 1.09 (t, *J* = 7.5 Hz , 3H, CH_3_), 2.80 (s, 3H, NCH_3_), 4.11 (q, *J* = 7.5 Hz, 2H, OCH_2_), 4.98 (s, 1H, ArCHNCH_3_), 7.15 (m, 2H, ArH), 7.33 (m, 3H, ArH) ppm; ^13^C-NMR: d 14.0 (CH_3_), 27.8 (NCH_3_), 61.1 (ArCHNCH_3_), 62.8 (CH_2_O), 113.0 (CCO), 127.7 (ArCH), 129.0 (ArCH), 134.9 (quart. ArC), 157.8 (C=O), 164.0 (C=O), 165.3 (COH) ppm; IR: 1691 (C=O), 1671 (C=O) cm^-1^; Anal. calcd. for C_14_H_15_NO_4_: C, 64.36; H, 5.79; N, 5.36. Found: C, 64.30; H, 5.81; N, 5.37.

*Ethyl 4-hydroxy-2-(4-methoxyphenyl)-1-methyl-5-oxo-2,5-dihydro-1H-pyrrole-3-carboxylate* (**1b**):Yield 28%, m.p 157-158 °C; ^1^H-NMR: d 1.09 (t, *J* = 6 Hz, 3H, CH_3_), 2.77 (s, 3H, NCH_3_), 3.77 (s, 3H, OCH_3_), 4.10 (q, *J* = 7.5 Hz, 2H, OCH_2_), 4.94 (s, 1H, ArCHNCH_3_), 6.84 (d, *J* = 9 Hz, 2H, ArH), 7.05 (d, *J* = 9 Hz, 2H, ArH), 9.08 (br s, 1H, OH) ppm; ^13^C-NMR: d 14.1 (CH_3_), 27.7 (NCH_3_), 55.4 (OCH_3_), 61.1 (ArCHNCH_3_), 62.3 (CH_2_O), 113.0 (CCO), 114.4 (ArCH), 126.6 (quart. ArC), 128.9 (ArCH), 157.5 (C=O), 160.1 (quart. ArC), 164.0 (C=O), 165.2 (COH) ppm; IR: 1675 (C=O), 1615 (C=O) cm^-1^; Anal. calcd. for C_15_H_17_NO_5 _: C, 61.85; H, 5.88; N, 4.81. Found: C, 61.83; H, 5.77; N, 4.82. 

*Ethyl 4-hydroxy-1-methyl-2-(4-methylphenyl)-5-oxo-2,5-dihydro-1H-pyrrole-3-carboxylate* (**1c**):Yield 31%, m.p 155-156 °C; ^1^H-NMR: d 1.12 (t, *J* = 7.5 Hz, 3H, CH_3_), 2.33 (s, 3H, NCH_3_), 2.79 (s, 3H, ArCH_3_), 4.06 (q, *J* = 7.5 Hz, 2H, OCH_2_), 4.95 (s, 1H, ArCHNCH_3_), 7.03 (d, *J* = 6 Hz, 2H, ArH), 7.13 (d, *J* = 9 Hz, 2H, ArH) ppm;^ 13^C-NMR: d 14.1 (CH_3_), 21.3 (ArCH_3_), 27.8 (NCH_3_), 61.1 (ArCHNCH_3_), 62.6 (OCH_2_), 113.0 (CCO), 127.6 (ArCH), 129.6 (ArCH), 131.8 (quart. ArC), 134.2 (quart. ArC), 138.2 (C=O), 157.7 (C=O), 165.3 (COH) ppm; IR: 1710 (C=O), 1665 (C=O) cm^-1^; Anal. calcd. for C_14_H_15_NO_4_: C, 65.44; H, 6.22; N, 5.09. Found: C, 65.39; H, 5.27. N, 5.08. 

*Ethyl 2-(4-chlorophenyl)-4-hydroxy-1-methyl-5-oxo-2,5-dihydro-1H-pyrrole-3-carboxylate* (**1d**):Yield 46%, m.p 215-216 °C; ^1^H-NMR: d 1.03 (t, *J* = 6.0 Hz, 3H, CH_3_), 2.60 (s, 3H, NCH_3_), 3.96 (q, *J* = 7.5 Hz, 2H, OCH_2_), 5.00 (s, 1H, ArCHNCH_3_), 6.70 (d, *J* = 9 Hz, 2H, ArH), 6.93 (d, *J* = 9 Hz, 2H, ArH), 9.20 (br s, 1H, OH), 11.18 (br s, 1H, OH) ppm;^ 13^C-NMR: 14.6 (CH_3_), 27.7 (NCH_3_), 60.0 (ArCHNCH_3_), 62.2 (CH_2_O), 112.2 (CCO), 116.0 (ArCH), 126.7 (quart. ArC), 129.2 (ArCH), 154.3 (C=O), 157.9 (C=O), 162.7 (quart. ArC), 165.0 (COH) ppm; IR: 1660 (C=O), 1613 (C=O) cm^-1^; Anal. calcd. for C_14_H_15_NO_5_: C, 60.64; H, 5.45; N, 5.05. Found: C, 60.61; H, 5.40; N, 4.99. 

### Syntheses of 5-(4-methoxyphenyl)-1-methylpyrrolidine-2,3-dione **(2b)**

2,3-Dioxopyrrolidine (**1b**, 3.38 g, 11.53 mmol) was dispersed in a 10% HCl solution (130 mL) and heated under reflux for 7 hours, during which it dissolved gradually to give a yellowish solution. The reaction mixture was then cooled and left standing overnight. Compound **2b** slowly precipitated out as a yellow solid, which was filtered, washed with water (5 mLx3) and ether (5 mLx3). After drying under reduced pressure **2b** was obtained (1.73 g, 68%), m.p. 156-157 °C; ^1^H-NMR: d 2.56 (dd,*J* = 7.5 Hz, 1H, CH_a_), 2.89 (s, 3H, NCH_3_), 3.13 (dd, *J* = 7.5 Hz, 1H, CH_b_), 3.81 (s, 3H, OCH_3_), 4.74 (d, *J* = 9 Hz, 2H, NCH_3_CHAr), 6.92 (d, *J* = 9 Hz, 2H, ArH), 7.11 (d, *J* = 6 Hz, 2H, ArH) ppm; ^ 13^C-NMR: d 29.7 (NCH_3_), 41.1 (CH_2_), 55.6 (OCH_3_), 58.1 (CH), 109.9 (quart. ArC), 115.1 (ArCH), 127.9 (ArCH), 130.3 (C=O), 160.3 (quart. ArC), 198.3 (C=O) ppm; IR: 1749 (C=O), 1702 (C=O) cm^-1^; Anal. calcd. for C_12_H_13_NO_3_: C, 65.74; H, 5.98; N, 6.39. Found: C, 65.70; H, 5.91; N, 6.43. 

### General procedures for the syntheses of hydrazone derivatives

Equimolar quantities (0.01 mol) of 5-(4-methoxyphenyl)-1-methylpyrrolidine-2,3-dione (**2b**) and the corresponding hydrazine salts were dissolved in warm ethanol. The reaction mixture was refluxed for 3 hours and then kept at room temperature overnight. The resulting solid was washed with ethanol and dried under reduced pressure to afford compounds **3a-c** (for **3d** the solvent of the product was removed under reduced pressure before being washed with ether). 

*(3E)-3-[2-(2,4-dinitrophenyl)hydrazinylidene]-5-(4-methoxyphenyl)-1-methylpyrrolidin-2-one* (**3a**): from **2b**, yield 65%, m.p 206-207 °C; ^1^H-NMR: d 2.79 (s, 3H, NCH_3_), 2.86 (dd, *J* = 7.5 Hz, 1H, CH_a_), 3.39 (dd, *J* = 7.5 Hz, 1H, CH_b_), 3.82 (s, 3H, OCH_3_), 4.70 (dd, *J* = 3 Hz, 1H, NCH_3_CHAr), 6.92 (d, *J* = 9 Hz, 2H, ArH), 7.15 (d, *J* = 9 Hz, 2H, ArH), 8.01 (d, *J* = 9 Hz, 1H, ArH), 8.29 (dd, *J* = 1.5 Hz, 1H, ArH), 9.11 (dd, *J* = 3 Hz, 1H, ArH), 10.94 (s br, 1H, NH) ppm;^ 13^C-NMR: d 28.6 (NCH_3_), 35.2 (CH_2_), 55.6 (OCH_3_), 60.9 (CH), 115.0 (ArCH), 115.9 (quart. ArC), 123.5 (ArCH), 127.9 (ArCH), 129.7 (ArCH), 141.0 (quart. ArC), 144.1 (quart. ArC), 144.7 (quart. ArC), 160.0 (quart. C=N), 160.3 (quart. ArC), 163.0 (C=O); IR: 3315 (NH), 1702 (C=O) cm^-1^; Anal. calcd. for C_18_H_17_N_5_O_6_: C, 54.14; H, 4.29; N, 17.54. Found: C, 54.05; H, 4.35; N, 17.60.

*4-{(2E)-2-[5-(4-methoxyphenyl)-1-methyl-2-oxopyrrolidin-3-ylidene]hydrazinyl}benzohydrazide* (**3b**): from **2b**, yield 55%, m.p 204-205 °C; ^1^H-NMR: d 2.61 (s, 3H, NCH_3_), 2.86 (dd, *J* = 7.5 Hz, 1H, CH_a_), 3.32 (dd, *J* = 7.5 Hz, 1H, CH_b_), 3.73 (s, 3H, OCH_3_), 4.32 (s br, 2H, NH_2_), 4.69 (d, *J* = 6 Hz, 1H, NCH_3_CHAr), 6.93 (d, *J* = 9 Hz, 2H, ArH), 7.15 (d, *J* = 9 Hz, 2H, ArH), 7.25 (d, *J* = 9 Hz, 1H, ArH), 8.08 (d, *J* = 9 Hz, 1H, ArH), 8.61 (s, 1H, ArH), 9.66 (s br, 1H, NH), 10.49 (s br, 1H, NH) ppm; ^13^C-NMR: d 28.9 (NCH_3_), 33.2 (CH_2_), 55.8 (OCH_3_), 59.1 (CH), 107.9 (quart. ArC), 115.1 (ArCH), 121.9 (quart. ArC), 128.5 (ArCH), 133.4 (ArCH), 137.6 (ArCH), 142.6 (ArCH), 148.0 (quart. ArC), 159.2 (quart. C=N), 159.7 (quart. ArC), 164.6 (C=O), 165.3 (C=O); IR: 3211 (NH), 1688 (C=O), 1601 (C=O) cm^-1^; Anal. calcd. for C_18_H_20_N_6_O_3_: C, 58.69; H, 5.47; N, 22.81. Found: C, 58.58; H, 5.30; N, 22.95.

*(3E)-3-[2-(1,3-benzothiazol-2-yl)hydrazinylidene]-5-(4-methoxyphenyl)-1-methylpyrrolidin-2-one* (**3c**): from **2b**, yield 67%, m.p 220- 221 °C; ^1^H-NMR: d 2.61 (s, 3H, NCH_3_), 2.86 (dd, *J* = 7.5 Hz, 1H, CH_a_), 3.32 (dd, *J* = 7.5 Hz, 1H, CH_b_), 3.73 (s, 3H, OCH_3_), 4.69 (d, *J* = 6 Hz, 1H, NCH_3_CHAr), 6.93 (d, *J* = 9 Hz, 2H, ArH), 7.13 (m, 3H, Ar-H), 7.28 (t, *J* = 7.5 Hz, 1H, ArH), 7.42 (d, *J* = 6 Hz, 1H, ArH), 7.77 (d, *J* = 6 Hz, 1H, ArH), 11.80 (s br, 1H, NH) ppm; ^13^C-NMR: d 29.0 (NCH_3_), 34.0 (CH_2_), 55.8 (OCH_3_), 59.1 (CH), 107.9 (quart. ArC), 115.1 (ArCH), 119.8 (quart. ArC), 122.4 (ArCH), 122.7 (ArCH), 126.7 (ArCH), 128.5 (ArCH), 133.1 (ArCH), 142.3 (quart. ArC), 159.8 (quart. ArC), 160.7 (quart. C=N), 164.8 (C=O), 169.7 (CNS); IR: 3061 (NH), 1680 (C=O) cm^-1^; Anal. calcd. for C_19_H_18_N_4_O_2_S: C, 62.28; H, 4.95; N, 15.29. Found: C, 62.15; H, 5.01; N, 15.15.

*(3E)-5-(4-methoxyphenyl)-1-methyl-3-[2-(pyridin-2-yl)hydrazinylidene]pyrrolidin-2-one* (**3d**): from **2b**, yield 57%, m.p 106-107 °C; ^1^H-NMR: d 2.71 (s, 3H, NCH_3_), 2.78 (dd, *J*= 7.5 Hz, 1H, CH_a_), 3.44 (dd, *J* = 7.5 Hz, 1H, CH_b_), 3.80 (s, 3H, OCH_3_), 4.77 (dd, *J* = 6 Hz, 1H, NCH_3_CHAr), 6.84 (t, *J* = 6 Hz, 1H, ArH), 6.96 (d, *J* = 9 Hz, 2H, ArH), 7.23 (d, *J* = 9 Hz, 2H, ArH), 7.38 (d, *J* = 9 Hz, 1H, ArH), 7.70 (t, *J* = 6 Hz, 1H, ArH), 8.10 (d, *J* = 3 Hz, 1H, ArH), 10.50 (s br, 1H, NH); ^13^C-NMR: d 28.9 (NCH_3_), 33.0 (CH_2_), 55.8 (OCH_3_), 59.0 (CH), 107.9 (quart. ArC), 115.1 (ArCH), 116.9 (ArCH), 128.4 (ArCH), 133.4 (ArCH), 138.8 (ArCH), 140.6 (ArCH), 148.2 (quart. ArC), 157.7 (quart. C=N), 159.7 (quart. ArC), 164.8 (C=O); IR: 3444 (NH), 1668 (C=O) cm^-1^; Anal. calcd. for C_17_H_18_N_4_O_2_ : C, 65.79; H, 5.85; N, 18.05. Found: C, 65.68; H, 5.90; N,18.17.
